# Effects of a health promotion intervention in the Mexican population with celiac disease

**DOI:** 10.1590/1980-220X-REEUSP-2024-0408en

**Published:** 2025-05-12

**Authors:** Itallo Carvalho-Gomes, Patricia Cruz-Bello, Maria de Lourdes García-Hernández, Olga Osorio-Murillo

**Affiliations:** 1 Universidad Autónoma del Estado de México, Facultad de Enfermería y Obstetricia, Toluca de Lerdo, México.; 2 Pontificia Universidad Javeriana Cali, Facultad de Enfermería, Departamento de Estudios Avanzados, Colombia.

**Keywords:** Celiac Disease, Health Promotion, Life Style, Adolescent, Young Adult, Doença Celíaca, Promoção da Saúde, Estilo de Vida, Adolescente, Adulto Jovem

## Abstract

**Objective:**

To assess the effect of a health promotion care model in adolescents and young adults with celiac disease.

**Method:**

A quasi-experimental study. A total of 136 people participated, who, after obtaining informed consent, received a virtual intervention in August and September 2023. The data were analyzed using the Wilcoxon test.

**Results:**

Regarding the celiac symptom index, statistically significant differences were found, with a large effect size, where pretest scores were higher than posttest scores (p < 0.001). Regarding lifestyle, it was found that health-promoting behaviors presented statistically significant differences, with a large effect size. Pretest scores were lower than posttest scores (p < .001).

**Conclusion:**

This groundbreaking study in Mexico demonstrated that health education significantly improves lifestyle and reduces symptoms in celiac patients who previously received limited attention to a gluten-free diet. It also highlights the crucial role of nurses as health educators in this field.

## RESUMO

**Objetivo:**

Avaliar o efeito de um modelo de atenção de promoção da saúde em adolescentes e adultos jovens com doença celíaca.

**Método:**

Estudo quase-experimental. Participaram 136 pessoas, às quais, mediante consentimento prévio e informado, foi aplicada uma intervenção virtualmente nos meses de agosto e setembro de 2023. Os dados foram analisados através do teste de Wilcoxon.

**Resultados:**

Em relação ao índice de sintomas celíacos foram encontradas diferenças estatisticamente significativas, com grande tamanho de efeito, onde os escores do pré-teste foram superiores aos escores do pós-teste (p < 0,001). Em relação ao estilo de vida, constatou-se que os comportamentos promotores de saúde apresentaram diferenças estatisticamente significativas, com grande tamanho de efeito. As pontuações do pré-teste foram inferiores às pontuações do pós-teste (p < 0,001).

**Conclusão:**

Este estudo inovador no México demonstrou que a educação em saúde melhora significativamente o estilo de vida e reduz os sintomas em pacientes celíacos, que anteriormente recebiam atenção limitada a uma dieta sem glúten. Além disso, destaca o papel crucial dos enfermeiros como educadores em saúde neste campo.

## INTRODUCTION

Celiac disease (CD) is an autoimmune disorder that manifests in people with a genetic predisposition and is characterized by triggering an immune system response to the presence of gluten in the body, generating a chronic inflammatory process of the intestinal mucosa and submucosa^(1)^. The most common symptoms are chronic diarrhea, constipation, pain, abdominal discomfort, etc. It can also cause a series of extra-gastrointestinal symptoms, such as anemia, depression, osteoporosis and cancer^(2)^.

CD is a pathology of the digestive and immune systems prevalent in the world’s population. Its incidence is estimated at 1% of the world’s population, affecting mainly women. Moreover, it is estimated that 1.7% of people with CD are symptomatic and between 0.75 and 1.2% are asymptomatic. In Mexico, it is estimated that 2.6% of the population has CD; however, only 0.9% has a diagnosis^(3,4)^.

The management of this pathology consists of a gluten-free diet and lifestyle changes. This involves adjusting daily habits to restore the intestinal mucosa and lead a life without complications triggered by the disease. However, for adolescents and young adults, it is a challenge to completely change their lifestyle, since these people in this age group already have their own lifestyle and, for this reason, end up not making any changes to their behavior and, consequently, suffer from the symptoms of CD^(5)^.

When referring to lifestyle, the present study refers to the set of behaviors and conducts that people develop and practice consciously and voluntarily throughout their lives^(6)^. In this way, lifestyle is considered one of the main determinants of the population’s health, since they are actions conditioned by biological, psychological and social traits, and are capable of assisting in the process of adopting healthy habits or, on the contrary, behaviors that are considered risk factors for health and a threat to individuals’ well-being^(7)^.

Health promotion, in turn, focuses on lifestyle changes, and adopts a behavioral and preventive health perspective. It brings the idea of empowerment by adopting an individual action approach and gives people and the community the opportunity to take control over their health, listing a set of fundamental principles for healthy living and highlighting the relevance of social participation and intersectoral incorporation for its practice and scope^(8,9)^.

Nola Pender’s Health Promotion Model served as a guide for the development of this intervention. This model emphasizes the importance of personal characteristics, such as health perception and self-control, as well as situational factors and health literacy. By considering these components, specific strategies were designed to promote individual participation and empowerment^(10)^.

Pender’s model aims to understand the reasons for human behavior related to health and guide people to adopt a healthy lifestyle and is based on two key points: the first highlights the relevance of cognitive processes in behavior change, considering that psychological aspects influence people’s behavior; and the second certifies that affirmative behavior is rational, justifying that the most significant element of achievement motivation is goal orientation^(11)^.

Literature review highlights the lack of studies investigating the application of the Nola Pender model in the context of CD. This gap highlights the importance of developing research that identifies the factors that influence the adoption of healthy behaviors by people with CD and tests the effectiveness of interventions based on this model. By filling this gap, it is possible to contribute to the development of more effective and personalized health promotion strategies for this population. For this reason, the objective of this research work is to evaluate the effect of a health promotion care model in adolescents and young adults with CD.

## METHOD

### Study Design

This is quantitative research with a quasi-experimental design of the pretest and posttest type. The research was carried out in a Mexican association for people with CD.

The intervention was structured in seven virtual sessions that addressed key topics for CD management. They began with an introduction to the disease and the importance of taking responsibility for health. Subsequently, gluten-free nutrition, physical activity, spiritual well-being and interpersonal relationships were discussed in depth. Moreover, strategies for managing stress were included. Each session included informative materials, videos and practical activities, and clear guidelines were established to optimize learning.

### Population

The research participants were the members of the association who agreed to participate. A non-probabilistic sample was selected. The sampling technique used was intentional. The sample consisted of 136 participants.

The inclusion criteria adopted were having a confirmed diagnosis of CD at the time of data collection, being between 15 and 35 years old, signing the Informed Consent Form and, in the case of minors, presenting the Informed Consent and Assent signed by their legal guardians and by the participant. The exclusion and elimination criteria were suffering from another chronic disease other than CD, such as non-celiac gluten sensitivity, and participants who dropped out of the research at any stage. Therefore, 16 participants were eliminated due to absence at any of the data collection stages.

### Data Collection

Data collection took place in three stages: application of pretest (diagnosis of reality); educational action (intervention in response to the demands that arose in the previous stage); and posttest (assessing the acquisition of knowledge, lifestyle changes and decrease in CD symptoms). All stages were carried out during 2022 and 2023, with an interval of six months between the first stage and the second and three months between the second stage and the third. All stages of the intervention were carried out through a web platform designed specifically for this purpose. This digital tool facilitated interaction between participants and professionals, allowing personalized monitoring of each individual. Once the intervention was completed, the web platform was deactivated.

The instruments used in the pretest and posttest were the Celiac Symptom Index (CSI), adapted in Mexico by Ramírez-Cervantes et al.^(12)^, and the Health Promoting Life Profile II (HPLP-II), translated and adapted to Spanish by Walker and Talley^(13)^. The CSI, divided into two categories (specific symptoms and general health), assesses individuals’ current health status with respect to CD. A higher score indicates greater severity of symptoms and a general deterioration in health. The HPLP-II, composed of six categories (health responsibility, physical activity, etc.), measures lifestyle health. The higher the score obtained, the healthier individuals’ lifestyle. Following the demands detected in pretest and guided by the Health Promotion Model^(14)^, The educational intervention addressed the following topics: understanding CD; taking responsibility for health; the importance of practicing physical activity; how to select and prepare gluten-free foods; the positive effect of spiritual development on life; the value of maintaining interpersonal relationships; and how to manage stress.

Once informed consent was obtained, participants received an email detailing the intervention dynamics. It consisted of seven virtual sessions, each lasting approximately 15 to 25 minutes, held every eight days.

Participants enjoyed complete autonomy to carry out the educational activities at their own pace and at the time that best suited their routine, always within the week corresponding to each topic. As proof of their participation, they sent a photograph of themselves in front of their computer or cell phone screen, confirming that they had completed the section. The photographs were sent to the email address of the intervention provider. The following material resources were needed: desktop computer, notebook or smartphone, internet access, sheets of paper and pens to take notes.

### Data Analysis and Processing

Data were organized in spreadsheets using the Statistical Package for the Social Sciences (SPSS Statistics) version 21, where they were analyzed using statistical techniques. The Shapiro-Wilk normality test was performed and, subsequently, the Wilcoxon test, due to the asymmetry of the data. To compare the results of pretest and posttest, concepts were created according to each participant’s score for each of the instruments used, such as CSI, with a single cut-off point where a score ≥ 35 suggested the presence of CD symptoms. For the HPLP-II, the score ranged from 52 to 208, and the higher the total score, the better the person’s lifestyle.

### Ethical Aspects

The research followed the ethical recommendations of the Regulations of the General Health Law on Health Research in Mexico, and was approved on July 1, 2022 by the *Universidad Autónoma del Estado de México* Research Ethics Committee, with Registration number 005/2022. All participants who agreed to participate in the study signed the Informed Consent Form.

## RESULTS

The study involved 136 people, all belonging to a non-profit association for people with CD in Mexico. Participants, mostly women, ranged in age from 18 to 35 years, with a median of 26.5 years and an interquartile range of 22 to 31 years. Regarding education, 88.2% had completed higher education; 6.6% had incomplete higher education and the rest had completed secondary education. As for occupation, 4.7% of the sample worked; 13.3% worked and studied, while 22% neither worked nor studied; and 52.9% of participants had access to social security.

### Celiac Symptom Index

Statistically significant differences with a large effect size were found in relation to celiac symptoms, where pretest scores (Mdn = 46.87; Range = 45.31) were higher than posttest scores (Mdn = 4.16; Range = 15.83) Z = −10.11, p <.001, Hedges G = 4.67 (Table 1).

**Table 1:** Comparison of CSI instrument scores at the two times (pretest and posttest) – Toluca de Lerdo, Edomex, Mexico, 2023.

	Time 1	Time 2	Z	p	Hedges G
	Mdn (range)	Mdn (range)			
**Celiac symptom index**	46.87 (45.31)	4.16 (15.83)	-10.11	<.001	4.67
**Specific symptoms**	2.90 (2.00)	.80 (8.00)	-7.87	<.001	1.35
**General health**	2.80 (3.20)	1.90 (6.11)	-4.15	<.001	0.37

Note: Mdn: median; Z: Wilcoxon test ratio value; p: statistical significance; Hedges G: effect size.

Source: own elaboration.

In the specific symptoms subcategory, statistically significant differences with a large effect size were found, where pretest scores (Mdn = 2.90; Range = 2.00) were higher than posttest scores (Mdn = .80; Range = 8.00) Z = −7.87, p<.001, Hedges G = 1.35 (Table 1).

Regarding the general health subcategory, despite the existence of statistically significant differences, where the pretest scores (Mdn = 2.80; Range = 3.20) were higher than the posttest scores (Mdn = 1.90; Range = 6.11) Z = −4.15, p < .001, the effect size was small (Hedges G = 0.37) (Table 1).

### Lifestyle

Regarding lifestyle, it was found that health-promoting behavior presented statistically significant differences with a large effect size. The pretest scores (Mdn = 52.90; Range = 55.48) were lower than the posttest scores (Mdn = 142.85; Range = 117.86) Z = −9.90, p < .001, G Hedges = 3.00 (Table 2).

**Table 2:** Comparison of the HPLP-II instrument scores at the two times (pretest and posttest) – Toluca de Lerdo, Edomex, Mexico, 2023.

	Time 1	Time 2	Z	p	Hedges G
	Mdn (range)	Mdn (range)			
**Health-promoting conduct**	52.90 (55.48)	142.85 (117.86)	-9.90	<.001	3.00
**Health responsibility**	2.44 (1.67)	18.11 (16.94)	-10.12	<.001	4.86
**Physical activity**	1.87 (2.75)	11.71 (14.06)	-10.11	<.001	3.76
**Nutrition**	2.33 (1.78)	14.96 (13.39)	-10.12	<.001	5.17
**Spiritual development**	2.88 (2.00)	16.53 (14.17)	-10.12	<.001	4.12
**Interpersonal relations**	3.22 (1.89)	16.53 (13.39)	-10.13	<.001	4.70
**Stress management**	2.25 (1.75)	14.84 (13.28)	-10.10	<.001	3.97

Note: Mdn: median; Z: Wilcoxon test ratio value; p: statistical significance; Hedges G: effect size.

Source: own elaboration.

Likewise, the subcategory responsibility in health presented statistically significant differences with a large effect size, where the pretest scores (Mdn = 2.44; Range = 1.67) were lower than the posttest scores (Mdn = 18.11; Range = 16.94) Z = −10.12, p < .001, G Hedges = 4.86 (Table 2).

The physical activity subcategory also presented statistically significant differences with a large effect size, where the pretest scores (Mdn = 1.87; Range = 2.75) were lower than the posttest scores (Mdn = 11.71; Range = 14.06) Z = −10.11, p < .001, Hedges G = 3.76 (Table 2).

In the nutrition component, the results showed that there were also statistically significant differences with a large effect size, where the pretest scores (Mdn = 2.33; Range = 1.78) were lower than the posttest scores (Mdn = 14.28; Range = 14.96) Z = −10.12, p < .001, Hedges G = 5.17 (Table 2).

Statistically significant differences with a large effect size were found in the spiritual development category, where the pretest scores (Mdn = 2.88; Range = 2.00) were lower than the posttest scores (Mdn = 16.53; Range = 14.17) Z = −10.12, p < .001, Hedges G = 4.12 (Table 2).

The interpersonal relationships subcategory also presented statistically significant differences with a large effect size, where the pretest scores (Mdn = 3.22; Range = 1.89) were lower than the posttest scores (Mdn = 16.53; Range = 13.39) Z = −10.13, p < .001, Hedges G = 4.70 (Table 2).

In turn, the stress management subcategory presented statistically significant differences with a large effect size, where the pretest scores (Mdn = 2.25; Range = 1.75) were lower than the posttest scores (Mdn = 14.84; Range = 13.28) Z = −10.10, p < .001, Hedges G = 3.97 (Table 2).

## DISCUSSION

In this study, the effectiveness of a health promotion intervention to change lifestyle and reduce symptoms of CD among Mexican adolescents and young adults was investigated.

Nowadays, with easy access to information, people have the opportunity to obtain any knowledge or clarification on the topic of their interest. However, there are some under-explored topics, such as health promotion for people with CD, as a comprehensive and detailed study focusing on assessing the needs of people with CD and designing a health promotion intervention for this specific audience was lacking. Therefore, this study was conducted using participants’ responses regarding their needs through the application of questionnaires.

Research on CD in Mexico and other countries has focused primarily on the prevalence of the disease. The only studies similar to this one found during a literature review were investigations that addressed the impact of education on raising awareness among people with CD^(15)^, in addition to an automated intervention through text messages with the sole objective of improving adherence to the gluten-free diet in celiac patients^(16)^ and a survey whose objective was to identify and validate educational needs to develop a self-care system for celiac patients^(17)^. Therefore, in the present research, a diagnosis was carried out to confirm the basic and mandatory educational elements that must be present in a health promotion intervention with the purpose of stimulating a change in the lifestyle of people with CD.

Regarding celiac symptoms, the pretest found that participants had symptoms of the disease and their general health was weak. During the intervention application, it was revealed, through the chat available on the intervention page, that some of them had had symptoms of the disease present for at least four weeks in a row, and according to reports, the lack of adequate knowledge about the disease, the lack of family support and the need to eat out due to work could be causing direct or cross contamination by gluten. These findings coincide with other research that mentions that celiac patients encounter many barriers to following treatment for the disease, which causes the emergence or persistence of symptoms^(18,19)^.

Complementing previous information, in the first session of the intervention, the topic of what CD is and how to treat it was addressed. The idea of this session was to bring participants closer to their reality, since, during the pretest phase, some demonstrated that they did not know enough about CD. Thus, after applying the posttest, it was possible to perceive that participants had better understood their situation, and that may have contributed to a reduction in these symptoms and an improvement in general health status.

Also, during the first phase, it was found that lifestyle was not healthy, which could explain the reason for the presence of symptoms of the disease in most of participants. Some explanations for not leading a healthy lifestyle, according to the subjects of the research, were the lack of knowledge about how to lead a healthy lifestyle, social pressure, fear of isolation, stress and anxiety caused by the feeling of exclusion. These findings confirm the results found in another study, which mentions that a more restricted lifestyle is a factor that contributes to emotional problems, due to feelings of deprivation and social avoidance, which can compromise the interpersonal relationships of these individuals^(20)^.

Thus, the intervention sessions taught and motivated participants to understand when it is time to seek professional help, as well as what types of physical activities were beneficial for their health, how to select and prepare food, avoiding cross contamination, the importance of always having direct and open contact with people without fear of informing them about CD and they were also taught relaxation and mentalization techniques to avoid stress and not feel excluded from society. After the application of the intervention focused on a healthy lifestyle, a significant improvement in their behavior was noted.

This work represents an innovation in the field of nursing, considering that the nurse is the patient’s first contact in the health sector and especially in primary care. It is important that the nurse has adequate knowledge about CD to help develop the necessary care and has the ability to transmit this care in a clear and concise manner, so that the patient can achieve therapeutic success^(21)^.

The absence of a dedicated physical research space, coupled with the virtual nature of all stages of the study, introduced certain limitations. The lack of face-to-face social interaction and the increased risk of technical issues may have negatively affected participant adherence, especially during the intervention phase. Moreover, the assessment of some variables, such as body language, was more complex in a virtual environment.

In order to optimize the results of future interventions, it is recommended that future research implement this intervention in person to maintain greater control over participants. It is also suggested that an intervention be designed for the family members of the person with CD, since, due to lack of knowledge about the disease, they do not offer the necessary support. Since CD can present genetic and cultural variations, this study, carried out with a Mexican population, contributes to the knowledge of the disease in this region. However, it is recommended that the research be replicated in South American countries to explore the diversity of clinical manifestations and factors associated with CD in different geographic and cultural contexts.

## CONCLUSION

Until now, there were no educational interventions to promote the health of the celiac population in Mexico, but this study brought an innovation in this field. With the results obtained, it can be concluded that it was necessary to take a direct look at celiac patients, since the concern of health professionals was focused solely on complying with the gluten-free diet and not on adopting a healthy lifestyle.

Thus, carrying out educational interventions to promote health in people with CD improves their lifestyle and reduces the symptoms of the disease, so the present study confirmed this hypothesis.

Another point to highlight is that nurses have relevance in acting as health educators, especially in health promotion programs and in the care of celiac patients. The importance of nursing in this field became evident, since nurses have a holistic vision and critical and reflective reasoning, having the ability to plan, implement and evaluate health education actions based on the execution of a situational diagnosis and the people involved in the process.
