# Supplementary material for: Effects of a health promotion intervention in the Mexican population with celiac disease
Source: Rev Esc Enferm USP. 2025 May 12;59:e20240408. doi: 10.1590/1980-220X-REEUSP-2024-0408en (PMC12070805; doi:10.1590/1980-220X-REEUSP-2024-0408en)
Supplement: Translation

# Translation: Efectos de una intervención de promoción de salud en población mexicana con enfermedad celíaca

**DOI:** 10.1590/1980-220X-REEUSP-2024-0408es

**Authors:** Itallo Carvalho-Gomes, Patricia Cruz-Bello, Maria de Lourdes García-Hernández, Olga Osorio-Murillo

**Affiliations:** 1 Universidad Autónoma del Estado de México, Facultad de Enfermería y Obstetricia, Toluca de Lerdo, México.; 2 Pontificia Universidad Javeriana Cali, Facultad de Enfermería, Departamento de Estudios Avanzados, Colombia.

**Keywords:** Enfermedad Celíaca, Promoción de la Salud, Estilo de Vida, Adolescente, Adulto Joven

## Abstract

**Objetivo:**

Evaluar el efecto de un modelo de cuidado de promoción de salud en adolescentes y adultos jóvenes con enfermedad celíaca.

**Método:**

Estudio cuasiexperimental. Participaron 136 personas, a quienes, previo consentimiento informado, se les aplicaron una intervención de manera virtual en los meses de agosto y septiembre de 2023. Los datos se analizaron a través de la prueba de Wilcoxon.

**Resultados:**

Con relación al índice de síntomas celíacos, se encontraron diferencias estadísticamente significativas, con un tamaño del efecto grande, donde las puntuaciones pretest fueron mayores que las puntuaciones postest (p < 0,001). En cuanto al estilo de vida, se encontraron que las conductas promotoras de la salud presentaron diferencias estadísticamente significativas, con un tamaño del efecto grande. Las puntuaciones pretest fueron inferiores a las puntuaciones del postest (p < .001).

**Conclusión:**

Este estudio innovador en México demostró que la educación en salud mejora significativamente el estilo de vida y reduce los síntomas en pacientes celíacos, quienes previamente recibían atención limitada a la dieta sin gluten. Además, resalta el papel crucial de los enfermeros como educadores de salud en este campo.

## INTRODUCCIÓN

La enfermedad celíaca (EC) es un trastorno autoinmune que se manifiesta en personas con propensión genética, y se caracteriza por provocar una respuesta del sistema inmunológico ante la presencia de gluten en el organismo, generando un proceso inflamatorio crónico de la mucosa y submucosa intestinal^(1)^. Los síntomas más comunes son diarrea crónica, estreñimiento, dolor, malestar abdominal, etc. También puede provocar una serie de síntomas extra gastrointestinales, como anemia, depresión, osteoporosis y cáncer^(2)^.

La EC es una patología del sistema digestivo e inmunológico prevalente en la población mundial. Su incidencia se estima en el 1% de la población mundial, afectando principalmente a mujeres. Además, se estima que el 1.7% de las personas con EC son sintomáticas y, entre el 0.75 y el 1.2%, son asintomáticas. En México, se estima que el 2.6% de la población tiene EC; sin embargo, solo el 0.9% tiene un diagnóstico^(3,4)^.

El manejo de esta patología consiste en una dieta libre de gluten y cambios en el estilo de vida. Esto implica el ajuste a los hábitos diarios para recuperar la mucosa intestinal y llevar una vida sin complicaciones desencadenadas por la enfermedad. Sin embargo, para adolescentes y adultos jóvenes, es un desafío cambiar completamente su estilo de vida, ya que estas personas en este grupo de edad ya tienen su propio estilo de vida y, por esta razón, terminan no realizando ningún cambio en su comportamiento y, en consecuencia, padecen los síntomas de la EC^(5)^.

Al mencionar estilo de vida, el presente estudio se refiere al conjunto de comportamientos y conductas que desarrollan y practican las personas de manera consciente y voluntaria a lo largo de su vida^(6)^. De esta manera, el estilo de vida se considera como uno de los principales determinantes de la salud de la población, ya que son acciones condicionadas por rasgos biológicos, psicológicos y sociales, y son capaces de coadyuvar en el proceso de adopción de hábitos saludables o, por el contrario, de conductas que sean consideradas factores de riesgo para la salud y una amenaza para el bienestar del individuo^(7)^.

A su vez, la promoción de la salud se centra en cambios en el estilo de vida, y adopta una perspectiva de salud conductual y preventiva. Trae la idea de empoderamiento al adoptar un enfoque de acción individual y brinda a las personas y a la comunidad la oportunidad de tomar control sobre su salud, enumerando un conjunto de principios fundamentales para una vida saludable y resaltando la relevancia de la participación social y la incorporación intersectorial para su práctica y alcance^(8,9)^.

El Modelo de Promoción de la Salud de Nola Pender sirvió como guía para el desarrollo de esta intervención. Este modelo enfatiza la importancia de las características personales, como la percepción de la salud y el autocontrol, así como los factores situacionales y los conocimientos sobre salud. Al considerar estos componentes, se diseñaron estrategias específicas para promover la participación y el empoderamiento de los individuos^(10)^.

El modelo de Pender pretende comprender el porqué de las conductas humanas relacionadas con la salud y orientar a las personas para que adopten un estilo de vida saludable y se basa en dos puntos clave: el primero destaca la relevancia de los procesos cognitivos en el cambio de conducta, considerando que los aspectos psicológicos influyen en el comportamiento de las personas; y el segundo certifica que la conducta afirmativa es racional, justificando que el elemento más significativo de la motivación para el logro es la orientación a las metas^(11)^.

La revisión de la literatura destaca la falta de estudios que investiguen la aplicación del modelo de Nola Pender en el contexto de la EC. Esta brecha resalta la importancia de desarrollar investigaciones que identifiquen los factores que influyen en la adopción de comportamientos saludables por parte de las personas con EC y prueben la efectividad de las intervenciones basadas en este modelo. Llenando este vacío, es posible contribuir al desarrollo de estrategias de promoción de la salud más efectivas y personalizadas para esta población. Por tal motivo, el objetivo de este trabajo de investigación es evaluar el efecto de un modelo de cuidado de promoción de salud en adolescentes y adultos jóvenes con EC.

## MÉTODO

### Tipo de Estudio

Se trata de una investigación cuantitativa con un diseño cuasiexperimental del tipo pretest y postest. La investigación se llevó a cabo en una asociación mexicana para personas celíacas.

La intervención se estructuró en siete sesiones virtuales que abordaron temas clave para la gestión de la EC. Se iniciaron con una introducción a la enfermedad y la importancia de asumir la responsabilidad en la salud. Posteriormente, se profundizó en la nutrición sin gluten, la actividad física, el bienestar espiritual y las relaciones interpersonales. Además, se incluyeron estrategias para manejar el estrés. Cada sesión contó con materiales informativos, videos y actividades prácticas, y se establecieron pautas claras para optimizar el aprendizaje.

### Población

Los participantes de la investigación fueron los miembros de la asociación que aceptaron participar. Se seleccionó una muestra no probabilística. La técnica de muestreo utilizada fue intencional. La muestra estuvo conformada de 136 participantes.

Los criterios de inclusión adoptados fueron tener diagnóstico confirmado de EC en el momento de la recolección de datos, tener entre 15 y 35 años, firmar el Formulario de Consentimiento Libre e Informado y, en el caso de menores de edad, presentar el Consentimiento Informado y Asentimiento firmado por sus tutores legales y por el participante. Los criterios de exclusión y eliminación fueron padecer otra enfermedad crónica distinta a la EC, por ejemplo sensibilidad al gluten no celíaca, y participantes que abandonaron la investigación en cualquier etapa. Por lo tanto, 16 participantes fueron eliminados por ausencia en alguna de las etapas de recolección de datos.

### Recolección de Datos

La recolección de datos se produjo en tres momentos: aplicación del pretest (diagnóstico de la realidad); acción educativa (intervención en respuesta a las demandas surgidas en la etapa anterior); y postest (evaluar la adquisición de conocimientos, cambios en el estilo de vida y disminución en los síntomas de la EC). Todas las etapas se realizaron durante los años 2022 y 2023, con un intervalo de seis meses entre la primera etapa y la segunda y de tres meses entre la segunda etapa y la tercera. Todas las etapas de la intervención se llevaron a cabo a través de una plataforma *web* diseñada específicamente para este propósito. Esta herramienta digital facilitó la interacción entre los participantes y los profesionales, permitiendo un seguimiento personalizado de cada individuo. Una vez finalizada la intervención, la plataforma *web* fue desactivada.

Los instrumentos utilizados en el pretest y postest fueron el *Celiac Symptom Index* (CSI), adaptado en México por Ramírez-Cervantes et al.^(12)^, y el *Health Promoting Life Profile II* (HPLP-II), traducido y adaptado al español por Walker y Talley^(13)^. El CSI, dividido en dos categorías (síntomas específicos y salud general), evalúa el estado de salud actual del individuo con respecto a la EC. Una puntuación más alta indica una mayor gravedad de los síntomas y un deterioro general de la salud. El HPLP-II, compuesto por seis categorías (responsabilidad en salud, actividad física, etc.), mide la salud del estilo de vida. Cuanto mayor sea la puntuación obtenida, más saludable será el estilo de vida del individuo. Siguiendo las demandas detectadas en el pretest y guiados por el Modelo de Promoción de la Salud^(14)^, la intervención educativa abordó los siguientes temas: conociendo la EC; asumiendo la responsabilidad con la salud; la importancia de practicar actividad física; cómo seleccionar y preparar alimentos sin gluten; el efecto positivo del desarrollo espiritual en la vida; el valor de mantener las relaciones interpersonales; y cómo manejar el estrés.

Una vez obtenido el consentimiento informado, los participantes recibieron un correo electrónico detallando la dinámica de la intervención. Esta constó de siete sesiones virtuales, con una duración aproximada de 15 a 25 minutos cada una, realizadas cada ocho días.

Los participantes disfrutaron de total autonomía para realizar las actividades educativas a su propio ritmo y en el horario que mejor se ajustara a su rutina, siempre dentro de la semana correspondiente a cada tema. Como comprobante de su participación, enviaron una fotografía suya frente a la pantalla de su computadora o celular, confirmando la realización de la sección. Las fotografías fueron enviadas a la dirección de correo electrónico del proveedor de la intervención. Se necesitaron los siguientes recursos materiales: computadora de escritorio, *notebook* o teléfono inteligente, acceso a internet, hojas de papel y bolígrafos para tomar notas.

### Análisis y Procesamiento de Datos

Los datos fueron organizados en hojas de cálculo mediante el *software Statistical Package for the Social Sciences* (SPSS *Statistics*), versión 21, donde fueron analizados mediante técnicas estadísticas. Se realizó la prueba de normalidad de Shapiro-Wilk y, posteriormente, la prueba de Wilcoxon, debido a la asimetría de los datos. Para comparar los resultados del pretest y postest, se crearon conceptos de acuerdo con la puntuación de cada participante para cada uno de los instrumentos utilizados, como CSI, con un punto de corte único donde una puntuación ≥ 35 sugería la presencia de síntomas de EC. Para el HPLP-II, la puntuación osciló entre 52 y 208, y cuanto mayor fuera la puntuación total, mejor era el estilo de vida de la persona.

### Aspectos Éticos

La investigación siguió las recomendaciones éticas del Reglamento de la Ley General de Salud en Materia de Investigación en Salud de México, y fue aprobada el 1 de julio de 2022 por el Comité de Ética en Investigación de la Universidad Autónoma del Estado de México, con número de Registro 005/2022. Todos los participantes que aceptaron participar en el estudio firmaron el Formulario de Consentimiento Libre e Informado.

## RESULTADOS

Participaron en el estudio 136 personas, todas pertenecientes a una asociación sin fines de lucro para personas celíacas en México. Los participantes, en su mayoría mujeres, tenían edades que oscilaban entre los 18 y los 35 años, con una mediana de 26,5 años y un rango intercuartil de 22 a 31 años. En cuanto a la formación, el 88.2% había completado los estudios superiores; el 6.6% tenía educación superior incompleta y los demás tenían educación secundaria completa. En cuanto a la ocupación, el 4.7% de la muestra trabajaba; el 13.3% trabajaba y estudiaba, mientras que el 22% no trabajaba ni estudiaba; y el 52.9% de los participantes tenía acceso a la seguridad social.

### Índice de Síntomas Celiacos

Se encontraron diferencias estadísticamente significativas con un tamaño de efecto grande en relación con los síntomas celíacos, donde las puntuaciones del pretest (Mdn = 46.87; Rango = 45.31) fueron mayores que las puntuaciones del postest (Mdn = 4.16; Rango = 15.83) Z = −10.11, p < .001, G Hedges = 4.67 (Tabla 1).

**Tabla 1:** Comparación de las puntuaciones del instrumento CSI en los dos tiempos (pretest y postest) – Toluca de Lerdo, Edomex, México, 2023.

	Tiempo 1	Tiempo 2	Z	p	G Hedges
	Mdn (rango)	Mdn (rango)			
**Índice de síntomas celiacos**	46.87(45.31)	4.16 (15.83)	-10.11	<.001	4.67
**Síntomas específicos**	2.90 (2.00)	.80 (8.00)	-7.87	<.001	1.35
**Salud general**	2.80 (3.20)	1.90 (6.11)	-4.15	<.001	0.37

Nota: Mdn: mediana; Z: valor de la razón prueba de Wilcoxon; p: significancia estadística; G Hedges: tamaño del efecto.

Fuente: elaboración propia.

En la subcategoría síntomas específicos, se encontraron diferencias estadísticamente significativas con un tamaño de efecto grande, donde las puntuaciones del pretest (Mdn = 2.90; Rango = 2.00) fueron mayores que las puntuaciones del postest (Mdn = .80; Rango = 8.00) Z = −7.87, p < .001, G Hedges = 1.35 (Tabla 1).

Con relación a la subcategoría salud general, a pesar de existir diferencias estadísticamente significativas, donde las puntuaciones del pretest (Mdn = 2.80; Rango = 3.20) fueron mayores que las puntuaciones del postest (Mdn = 1.90; Rango = 6.11) Z = −4.15, p < .001, el tamaño del efecto fue pequeño (G Hedges = 0.37) (Tabla 1).

### Estilo de Vida

Con relación al estilo de vida, se encontró que la conducta promotora de salud presentó diferencias estadísticamente significativas con un tamaño de efecto grande. Las puntuaciones del pretest (Mdn = 52.90; Rango = 55.48) fueron menores que las puntuaciones del postest (Mdn = 142.85; Rango = 117.86) Z = −9.90, p < .001, G Hedges = 3.00 (Tabla 2).

**Tabla 2:** Comparación de las puntuaciones del instrumento HPLP-II en los dos tiempos (pretest y postest) – Toluca de Lerdo, Edomex, México, 2023.

	Tiempo 1	Tiempo 2	Z	p	G Hedges
	Mdn (rango)	Mdn (rango)			
**Conducta promotora de salud**	52.90 (55.48)	142.85 (117.86)	-9.90	<.001	3.00
**Responsabilidad en salud**	2.44 (1.67)	18.11 (16.94)	-10.12	<.001	4.86
**Actividad física**	1.87 (2.75)	11.71 (14.06)	-10.11	<.001	3.76
**Nutrición**	2.33 (1.78)	14.96 (13.39)	-10.12	<.001	5.17
**Desarrollo espiritual**	2.88 (2.00)	16.53 (14.17)	-10.12	<.001	4.12
**Relaciones interpersonales**	3.22 (1.89)	16.53 (13.39)	-10.13	<.001	4.70
**Manejo delestrés**	2.25 (1.75)	14.84 (13.28)	-10.10	<.001	3.97

Nota: Mdn: mediana; Z: valor de la razón prueba de Wilcoxon; p: significancia estadística; G Hedges: tamaño del efecto.

Fuente: elaboración propia.

Asimismo, la subcategoría responsabilidad en salud presentó diferencias estadísticamente significativas con un tamaño de efecto grande, donde las puntuaciones del pretest (Mdn = 2.44; Rango = 1.67) fueron menores que las puntuaciones del postest (Mdn = 18.11; Rango =16.94) Z = −10.12, p < .001, G Hedges = 4.86 (Tabla 2).

La subcategoría actividad física también presentó diferencias estadísticamente significativas con un tamaño de efecto grande, donde las puntuaciones del pretest (Mdn = 1.87; Rango =2.75) fueron menores que las puntuaciones del postest (Mdn = 11.71; Rango = 14.06) Z = −10.11, p < .001, G Hedges = 3.76 (Tabla 2).

En el componente de nutrición, los resultados mostraron que también hubo diferencias estadísticamente significativas con un tamaño de efecto grande, donde las puntuaciones del pretest (Mdn = 2.33; Rango = 1.78) fueron menores que las puntuaciones del postest (Mdn = 14.28; Rango = 14.96) Z = −10.12, p < .001, G Hedges = 5.17 (Tabla 2).

Se encontraron diferencias estadísticamente significativas con un tamaño de efecto grande en la categoría desarrollo espiritual, donde las puntuaciones del pretest (Mdn = 2.88; Rango = 2.00) fueron menores que las puntuaciones del postest (Mdn = 16.53; Rango = 14.17) Z = −10.12, p < .001, G Hedges = 4.12 (Tabla 2).

La subcategoría relaciones interpersonales también presentó diferencias estadísticamente significativas con un tamaño de efecto grande, donde las puntuaciones del pretest (Mdn = 3.22; Rango = 1.89) fueron menores que las puntuaciones del postest (Mdn = 16.53; Rango = 13.39) Z = −10.13, p < .001, G Hedges = 4.70 (Tabla 2).

A su vez, la subcategoría manejo del estrés presentó diferencias estadísticamente significativas con un tamaño de efecto grande, donde las puntuaciones del pretest (Mdn = 2.25; Rango = 1.75) fueron menores que las puntuaciones del postest (Mdn = 14.84; Rango = 13.28) Z = −10.10, p < .001, G Hedges = 3.97 (Tabla 2).

## DISCUSIÓN

En este estudio, se investigó la efectividad de una intervención de promoción de la salud para cambiar el estilo de vida y reducir los síntomas de la EC entre adolescentes y adultos jóvenes mexicanos.

Hoy en día, con un fácil acceso a la información, las personas tienen la oportunidad de obtener cualquier conocimiento o aclaración sobre el tema de su interés. Sin embargo, hay algunos temas poco explorados, como la promoción de la salud de las personas celíacas, ya que faltaba un estudio completo y detallado que se centrara en la evaluación de las necesidades de las personas celíacas y en el diseño de una intervención de promoción de la salud para este público específico. Por lo tanto, este estudio se realizó mediante las respuestas de los participantes en cuanto a sus necesidades a través de la aplicación de cuestionarios.

Las investigaciones sobre EC en México y otros países se han enfocado principalmente en la prevalencia de la enfermedad. Los únicos estudios similares a este encontrados, durante una revisión de la literatura, fueron investigaciones que abordaron el impacto de la educación en la concientización de las personas con EC^(15)^, además de una intervención automatizada a través de mensajes de texto con el único objetivo de mejorar la adherencia a la dieta sin gluten en personas celíacas^(16)^ y una encuesta cuyo objetivo fue identificar y validar necesidades educativas para desarrollar un sistema de autocuidado para pacientes celíacos^(17)^. Por lo tanto, en la presente investigación, se realizó un diagnóstico para confirmar los elementos educativos básicos y obligatorios que deben estar presentes en una intervención de promoción de la salud con el propósito de estimular un cambio en el estilo de vida de las personas con EC.

Con respecto a los síntomas celíacos, se encontró en el pretest que los participantes presentaban síntomas de la enfermedad y su salud general presentaba debilidad. Durante la aplicación de la intervención, se dio a conocer, a través del chat disponible en la página de la intervención, que algunos de ellos habían tenido síntomas de la enfermedad presentes durante al menos cuatro semanas seguidas, y según relatos, la falta de conocimiento adecuado sobre la enfermedad, la falta del apoyo de la familia y la necesidad de comer fuera de casa debido al trabajo podrían estar ocasionando contaminación directa o cruzada por gluten. Estos hallazgos coinciden con otras investigaciones quienes mencionan que el paciente celiaco encuentra muchas barreras para seguir el tratamiento de la enfermedad, lo que ocasiona el surgimiento o persistencia de los síntomas^(18,19)^.

Complementando la información previa, en la primera sesión de la intervención, se abordó lo qué es la EC y cómo tratarla. La idea de esta sesión fue acercar al participante a su realidad, ya que, durante la fase pretest, algunos demostraron que no conocían lo suficiente sobre la EC. Así, tras aplicar el postest se pudo percibir que los participantes habían comprendido mejor su situación, y eso pudo haber contribuido a una reducción de estos síntomas y una mejoría en el estado de salud general.

Asimismo, durante la primera fase, se encontró que el estilo de vida no era saludable, lo que podría explicar el motivo de la presencia de síntomas de la enfermedad en la mayoría de los participantes. Algunas explicaciones para no llevar un estilo de vida saludable, según los sujetos de la investigación, fueron la falta de conocimiento sobre cómo llevar un estilo de vida saludable, la presión social, el miedo al aislamiento, el estrés y la ansiedad provocados por el sentimiento de exclusión. Estos hallazgos confirman los resultados encontrados en otro estudio, el cual menciona que un estilo de vida más restringido es un factor que contribuye a los problemas emocionales, debido a sentimientos de privación y evitación social, que pueden comprometer las relaciones interpersonales de estos individuos^(20)^.

Así, las sesiones de intervención enseñaron y motivaron a los participantes a comprender cuándo es el momento de buscar ayuda profesional, así como qué tipos de actividades físicas eran beneficiosas para su salud, cómo seleccionar y preparar los alimentos, evitando la contaminación cruzada, la importancia de tener siempre un contacto directo y abierto con las personas sin miedo a informarles sobre la EC y también se les enseñaron técnicas de relajación y mentalización para evitar el estrés y no sentirse excluidos de la sociedad. Después de la aplicación de la intervención enfocada en un estilo de vida saludable, se notó una mejora significativa en su comportamiento.

Este trabajo supone una innovación en el campo de la enfermería, considerando que la enfermera es el primer contacto del paciente en el sector sanitario y especialmente en la atención primaria. Es importante que la enfermera tenga conocimientos adecuados sobre EC para ayudar a desarrollar los cuidados necesarios y tenga la capacidad de transmitir estos cuidados de forma clara y concisa, para que el paciente pueda alcanzar el éxito terapéutico^(21)^.

La ausencia de un espacio físico dedicado a la investigación, sumada a la naturaleza virtual de todas las etapas del estudio, introdujo ciertas limitaciones. La falta de interacción social cara a cara y el mayor riesgo de problemas técnicos pudieron haber afectado negativamente la adherencia de los participantes, especialmente durante la fase de intervención. Además, la evaluación de algunas variables, como el lenguaje corporal, resultó más compleja en un entorno virtual.

Con el fin de optimizar los resultados de futuras intervenciones, se recomienda que futuras investigaciones implementen esta intervención de manera presencial para mantener un mayor control sobre los participantes. También se sugiere diseñar una intervención para los familiares de la persona con EC, ya que, por desconocimiento sobre la enfermedad, no ofrecen el apoyo necesario. Dado que la EC puede presentar variaciones genéticas y culturales, este estudio, realizado con una población mexicana, contribuye al conocimiento de la enfermedad en esta región. Sin embargo, se recomienda replicar la investigación en países sudamericanos para explorar la diversidad de manifestaciones clínicas y factores asociados a la EC en diferentes contextos geográficos y culturales.

## CONCLUSIÓN

Hasta el momento, no existían intervenciones educativas para promover la salud de la población celíaca en México, pero este estudio trajo una innovación en este campo. Con los resultados obtenidos, se puede concluir que era necesario realizar una mirada directa al paciente celíaco, ya que la preocupación de los profesionales sanitarios se centraba únicamente en cumplir la dieta sin gluten y no en adoptar un estilo de vida saludable.

De esta forma, realizar intervenciones educativas para promover la salud en personas celíacas mejora su estilo de vida y reduce los síntomas de la enfermedad, por lo que el presente estudio confirmó esta hipótesis.

Otro punto que resaltar es que los enfermeros tienen relevancia en actuar como educadores en salud, especialmente en programas de promoción de la salud y en la atención al paciente celíaco. Se hizo evidente la importancia de la enfermería en este campo, ya que las enfermeras tienen una visión holística y un razonamiento crítico y reflexivo, teniendo la capacidad de planificar, implementar y evaluar acciones de educación en salud a partir de la ejecución de un diagnóstico situacional y de las personas involucradas en el proceso.
